# Exogenous spermidine alleviates diabetic cardiomyopathy via suppressing reactive oxygen species, endoplasmic reticulum stress, and Pannexin-1-mediated ferroptosis

**DOI:** 10.17305/bb.2022.8846

**Published:** 2023-10-01

**Authors:** Jian Sun, Jiyu Xu, Yong Liu, Yitong Lin, Fengge Wang, Yue Han, Shumin Zhang, Xiaoyan Gao, Changqing Xu, Hui Yuan

**Affiliations:** 1 School of Basic Medical Sciences, Mudanjiang Medical University, Mudanjiang, China; 2 School of Medical Imaging, Mudanjiang Medical University, Mudanjiang, China; 3 Animal Research Institute, Research Department, Mudanjiang Medical University, Mudanjiang, China; 4 The Fifth Affiliated Hospital of Southern Medical University, Guangzhou, China; 5 School of Stomatology, Mudanjiang Medical University, Mudanjiang, China; 6 Department of Pathophysiology, Harbin Medical University, Harbin, China

**Keywords:** Diabetic cardiomyopathy (DCM), Spermidine (SPD), ferroptosis, Pannexin-1, P2X7

## Abstract

Diabetic cardiomyopathy (DCM) is a serious complication and death cause of diabetes mellitus (DM). Recent cardiology studies suggest that spermidine (SPD) has cardioprotective effects. Here, we verified the hypothesis of SPD’s protective effects on DCM. Therefore, db/db mice and primary neonatal mouse cardiomyocytes were used to observe the effects of SPD. Immunoblotting showed that ornithine decarboxylase (ODC) and SPD/spermine N1-acetyltransferase (SSAT) were downregulated and upregulated in the myocardium of db/db mice, respectively. We found that diabetic mice showed cardiac dysfunction in 12 weeks. Conversely, exogenous SPD could improve cardiac functions and reduce the deposition of collagens, myocardial damage, reactive oxygen species (ROS) levels, and endoplasmic reticulum stress (ERS) in diabetic mouse hearts. Our results also demonstrated that cardiomyocytes displayed ferroptosis and then activated Pannexin-1 expression, which resulted in the increase of the extracellular adenosine triphosphate (ATP). Subsequently, increased ATP as a paracrine molecule combined to purinergic receptor P2X7 to activate ERK1/2 signaling pathway in cardiomyocytes and activated NCOA4-mediated ferroptinophagy to promote lipid peroxidation and ferroptosis. Interestingly, SPD could reverse these molecular processes. Our findings indicate an important new mechanism for DCM and suggest that SPD has potential applicability to protect against deterioration of cardiac function with DCM.

## Introduction

Diabetes mellitus (DM) is a metabolic disease, divided into type 1 and type 2. The former is usually induced by congenital pancreatic islet dysfunction, and the latter with the higher incidence is mostly caused by insulin resistance [1]. DM can cause persistent hyperglycemia and metabolic disorders, and then can lead to the impairment of tissues and organs, particularly damaging the cardiovascular system [2, 3]. DCM is an impairment of ventricular diastolic and systolic function induced by no coronary artery disease, which is distinct from cardiomyopathy caused by coronary heart disease and hypertension [4]. The pathogenesis of DCM is complex and inconclusive, and the underlying mechanisms are all related to the interaction of multiple damage factors of hyperglycemia, which affect the heart structural function. It may be related to mitochondrial dysfunction in cardiomyocytes, abnormal activation of cardiac fibroblasts, imbalance of redox system, and extracellular matrix (ECM) [5, 6]. However, the exact mechanism of DCM remains unknown. Therefore, finding accurate and effective therapeutic target drugs is the priority.

Polyamines (PAs) are small branched chain cationic molecule derived from a linear amino acid, including spermine (SP), spermidine (SPD), and putrescine (PU), and are widely distributed in all types of mammalian cells [7]. Intracellular levels of SPD are maintained and tightly controlled by enzymes that catalyze rate-limiting steps of their biosynthesis by ornithine decarboxylase (ODC) and catabolism by SPD/SP N1-acetyltransferase (SSAT) [8, 9]. SPD participates in various biological processes of organism, including regulation of DNA synthesis, cell cycle, cell proliferation and differentiation, aging, endoplasmic reticulum stress (ERS), oxidative stress, and ion channel switching, and also has anti-apoptosis [10], anti-inflammatory [11], and antioxidant and induction of autophagy [12]. Some studies highlight the effects of SPD for aging via improving mitochondrial biogenesis and function [7, 13, 14], and recent researches demonstrate that SPD can prevent heart injury by inhibiting oxidative stress and ERS [8]. However, it is unclear whether the administration of exogenous SPD to diabetic heart can reduce myocardial damage.

Our team, and others, recently corroborated that SPD and SP levels were decreased in diabetic rats [15, 16], and treatment of exogenous SP can protect cardiomyocytes to attenuate rat diabetic cardiomyopathy (DCM) [17, 18]. However, whether SPD can mitigate DCM has not yet been elucidated. Recent research shows that ferroptosis is involved in diabetes myocardial injury and Pannexin-1 expression is a key factor. Hence, in this study, we hypothesized SPD exerted beneficial effects on DCM according to the above mechanisms [19, 20]. The therapeutic implication of SPD in DCM development was evaluated in db/db mouse models, the mechanism of SPD was determined using cardiomyocytes undergoing high glucose model.

## Materials and methods

### Experimental animals

Homozygous eight-week-old male db/db mice (22 ± 0.5 g) on a C57BL/6 background were provided by the Animal Research Institute of Mudanjiang Medical University (MMU), and the study was approved by the MMU Medical Science Ethics Committee. All mice were maintained on a 12-h light/dark cycle and fed with a standard chow and clean water ad libitum. The mice were randomly divided into four groups (*n* ═ 8 per group): (1) Wild-type (WT) group: eight-week-old male C57BL/6 mice were injected with stroke-physiological saline solution buffer (SPSS); (2) WT+SPD group: C57BL/6 mice intraperitoneal injection of SPD (Cat NO. 124-20-9, Sigma, St. Louis, MO, USA) dissolved in SPSS (10 mg/kg); (3) Type 2 diabetes (T2D) group: db/db mice; (4) T2D+SPD group: db/db mice intraperitoneal injection of SPD (10 mg/kg in SPSS), the dosing interval of animals in each group was every other day. All mice in the four groups were sacrificed in week 12 and determined relevant experimental indexes according to the protocols.

### Cell culture

Primary neonatal mouse cardiomyocytes were isolated from the hearts of 1–3-day old C57BL/6 mice. In brief, in a sterile operating table, the hearts were quickly removed, immediately placed in pre-chilled D-hanks solution. All hearts in the dish were washed and transferred to a 15 ml centrifuge tube, added 3 ml D-Hanks and 5 ml 0.25% trypsin, sealed, and placed on a shaker at 4 ^∘^C overnight. The next day, added 5 ml Dulbecco’s Modified Eagle Medium (DMEM) containing 10% fetal bovine serum and 1% penicillin and streptomycin, and then abandoned DMEM, added 7 ml type II collagenase (0.08%, prepared with serum-free DMEM). Then put it on a shaker, 37 ^∘^C, 160 r/min, 10 min, and collected the supernatant DMEM in a 50 ml centrifuge tube (store at 4 ^∘^C). The abovementioned process was repeated 3–5 times until all hearts were completely digested. The cells in the 50 ml centrifuge tube were collected by centrifugation at 4 ^∘^C, 800 r/min, 5 min. Subsequently, added the medium and mixed well, inoculated in Petri dishes, and 2 h of incubation, the unattached cells were cardiomyocytes. The cardiomyocytes were planted in another Petri dish and maintained at 37 ^∘^C in a 5% CO_2_ humidified incubator. The media of cells was changed three times per week. Cardiomyocytes were treated with high glucose (HG, 40 mM), SPD (10 µM), Erastin (0.25 µM, Cat NO. 571203-78-6, Sigma), P2X7 specific inhibitor (3 µM, A-438079, Cat NO. 899431-18-6, Selleck), LY3214996 (20 µM, Cat NO. 1951483-29-6, Selleck), according to the experiment protocol.

### Serum measurements

Blood glucose was measured from the mouse tail vein blood by a blood glucose meter (ACCU-CHEK, Roche, Germany). In week 12, blood samples taken from the medial canthal vein were centrifuged and serum was used to detect relevant indicators. Serum insulin levels were determined by ELISA kit (Cat NO.TW-JL11459, Tongwei, Shanghai, China). Serum levels of triacylglycerol (TG) and total cholesterol (TC) were analyzed using a standard biochemistry panel (Cat NO. S0112, S0321, Beyotime, China). Lactate dehydrogenase (LDH), creatine kinase isoenzyme (CK-MB), and cardiac troponin-I (cTnI) in the blood serum were measured by using commercially available kits (Cat NO. A020-1, H197-1, H149-2, Jiancheng Institute of Bioengineering, Nanjing, China). All kits were operated according to the manufacturer’s instructions.

### Glucose tolerance tests

Mice were intraperitoneally injected with D-glucose (2 g/kg mass). Tail vein blood was collected, and blood glucose was detected using a glucometer (ACCU-CHEK, Roche, Germany).

### Iron measurements

Heart tissue and cardiomyocytes were homogenized with phosphate-buffered saline (PBS) and the supernatant was collected after centrifugation. The iron levels of tissue and cells were detected by the Iron Assay Kit (Cat NO. ab83366, Abcam) according to the manufacturer’s instructions.

### GPX-4 activity and GSH level measurements

 As described method in the previous protocol [21], glutathione peroxidase 4 (GPX-4) activity was detected by using phosphatidylcholine hydroperoxide as a substrate. Total glutathione (GSH) levels of the mouse heart tissue were determined using GSH/GSSG Assay Kit (Cat NO. S0053, Beyotime, Shanghai, China).

### Histology analysis

 After anesthesia, the heart was quickly excised and washed with pre-cooled PBS buffer. The cardiac tissue was fixed in 10% buffered paraformaldehyde, embedded in paraffin, sliced at 4 mm, and used for morphological observation. Hematoxylin and eosin staining (HE), Masson trichrome staining, and Sirius red staining were conducted according to the dyeing protocol, tissue-stained sections were observed using computer-assisted color image analysis system (Leica Microscope DM2700M, Gemany).

### Echocardiographic analysis

A Vivid 7 Dimension echocardiography machine was used to assess cardiac function and dimensions (Mylab Delta-vet, Esaote, Italy). All the mice were anesthetized with 2% isoflurane to perform echocardiography. Left ventricular ejection fraction (EF), left ventricular fractional shortening (FS), left ventricular internal dimension systole (LVIDs), and left ventricular internal dimension diastole (LVIDd) were measured.

### Transmission electron microscopy (TEM)

The ultrastructural analysis was performed as previously described [22]. Briefly, heart tissue was fixed with 2.5% glutaraldehyde overnight at 4 ^∘^C, fixed in 1% osmium tetroxide for 2 h. Subsequently, heart tissue was dehydrated using a graded ethanol series, embedded in epoxy resin, and observed using a H-7650 transmission electron microscope (Hitachi, Japan).

### Bioinformatic analysis

Heart samples were lysed, and trypsin digested according to LC-MS/MS analysis procedure. Mass spectral data were retrieved using Proteome Discoverer (v2.4.1.15). The searching database is Mus_musculus_10090_SP_20201214.fasta (17063 sequences). A heat map was drawn by using the “gplots” R-package. Kyoto Encyclopedia of Genes and Genomes (KEGG) enrichment analysis, enrichment of gene ontology (GO) biological process, cellular component, and molecular function terms were analyzed by using DAVIDs Functional Annotation Chart tool (Version 6.8) [23, 24].

### Mitochondrial membrane potential (**Δ ψ**m) analysis

Δ ψm was determined using a JC1 Mitochondrial Membrane Potential Assay Kit (Cat NO. C2003S, Beyotime, Shanghai). In brief, after treatment with HG and SPD, cardiomyocytes were incubated with 10 µM JC1 for 30 min at 37 ^∘^C in the dark. The images were then taken with a laser scanning confocal microscope (FV1000-IX81, OLYMPUS) at an excitation 488 nm wavelength and emission 530 nm wavelengths and analyzed with Image J 1.46 software. Red fluorescence was JC1 aggregate, green fluorescence was JC1 monomer in the mitochondria.

### Reactive oxygen species (ROS) staining

The level of reactive oxygen species (ROS) was assessed through the use of dihydroethidium (DHE) (Cat NO. S0063, Beyotime, Shanghai, China) staining on freshly frozen tissue sections. A solution of 5 µM DHE dissolved in DMSO was added to the sections of mouse heart tissue after being diluted in PBS. The sections were then incubated in darkness at a temperature of 37 ^∘^C for 30 min, followed by two rinses with cold PBS. Photos were captured immediately, and the fluorescence density was measured using Image J.

### ROS and lipid ROS measurements

Oxidative stress was detected using an ROS kit (Cat NO. S0033S, Beyotime, Shanghai). Briefly, diluted DCFH-DA with serum-free medium at 1:1000 to make the final concentration 10 µM, removed the cardiomyocytes culture medium and added an appropriate volume of diluted DCFH-DA, incubated in 37 ^∘^C condition for 20 min. The cells were washed three times with serum-free cell culture medium to fully remove DCFH-DA, and then used laser scanning confocal microscope (FV1000-IX81, OLYMPUS) at an excitation 488 nm wavelength and emission 525 nm wavelength to take photograph and analyzed with Image J 1.46 software. Lipid ROS was detected by using BODIPY 581/591 C11 fluorescent probe (Cat NO. D3861, Invitrogen) as the previous protocol [25].

### Immunofluorescence analysis

Frozen sections of heart tissue were washed, fixed, and permeabilized. Subsequently, the slices were immunolabelled with specific primary antibodies (Pannexin-1 at 1:50 ratio) overnight at 4 ^∘^C. After rinsing, the slices were incubated with corresponding fluorescent-conjugated secondary antibody (Cat NO. AS011, goat anti-Rabbit IgG, diluted 1:500, abclonal, Wuhan), stained nuclei with DAPI, and observed using laser scanning confocal microscope (FV1000-IX81, OLYMPUS).

### Adenosine triphosphate (ATP) analysis

The concentration of adenosine triphosphate (ATP) in cardiomyocytes lysate was measured using ATP Assay Kit (Cat NO. S0026, Beyotime Biotechnology, Shanghai), and intracellular ATP was measured according to the instructions provided by the manufacturer.

To detect the concentration of extracellular ATP, the culture medium was processed by a special method [26]. Briefly, cardiomyocytes were treated with different experimental protocol for 24 h, the medium was collected and ATP release was measured by the luciferin–luciferase assay with an ATP Assay Kit (Cat NO. S0027, Beyotime Biotechnology, Shanghai), following the manufacturer’s instructions [17].

### siRNA transfection

The cardiomyocytes were seeded in a 35 mm dish treated with Pannexin-1 short interfering RNAs (Cat NO. sc-61287, Santa Cruz Biotechnology), following the experimental protocol. In brief, cells were transfected by siRNA using PolyJetTM (Signagen), Con-siRNA, and Pannexin-1–siRNA and the transfection reagent was incubated for 12 h with serum-free medium to inhibit the relevant protein expression according to the manufacturer’s instructions and replaced with normal medium containing 10% FBS to continue to incubate for 12 h, and then the next experiment could be processed.

### Western blot analysis

The mouse heart tissue and cardiomyocytes were homogenized in 0.5 ml of RIPA (1:1000 phenylmethanesulfonylfluoride, PMSF, Cat NO. R0010, Solarbio Science, Beijing) buffer using small tubes and vibrated for every 10 min for 5 s at 4 ^∘^C, the above process repeats four times. Solubilized proteins were centrifugated at 13,500 rpm for 25 min, the supernatant was then collected. The protein concentration of each sample was quantified using the BCA Protein Assay kit (Cat NO. P0011, Beyotime, Shanghai). Protein lysates of each group were separated by electrophoresis with SDS-PAGE and electro-transferred onto a PVDF membrane (Millipore). Non-specific proteins on membranes were blocked with 5% non-fat dried milk for 2 h at room temperature, the membranes were incubated overnight with the following primary antibodies (at a 1:1000 dilution, 4 ^∘^C): P2X7, ACSL-4, GPX-4, FTH-1, HO-1, NCOA-4, SSAT (Cat NO. A10511, A6826, A1933, A1144, A1346, A5695, A2506, ABclonal Technology, Wuhan, China); Prdx-1, TfR-1, p-p38, t-p38, p-JNK, t-JNK, GRP94, GRP78, ATF-4, Chop (Cat NO. 15816-1-AP, 10084-2-AP, 28796-1-AP, 14064-1-AP, 80024-1-RR, 24164-1-AP, 60012-2-Ig, 11587-1-AP, 10835-1-AP, 15204-1-AP, Proteintech, Wuhan, China); and ODC, ERK1/2, p-ERK1/2, Pannexin-1, β-actin and β-tubulin (Cat NO. sc-390366, sc-81457, sc-136521, sc-515941, sc-69879, sc-166729 Santa Cruz Biotechnology). And then the membranes were incubated with anti-mouse/anti-rabbit IgG antibody (Cat NO. AS003, AS029, ABclonal Technology, Wuhan, China) at a 1:10000 dilution for 1 h at room temperature. The specific complex was determined by an enhanced chemiluminescent (ECL) kit (NO. MA0186, Meilun, Dalian, China) and a multiplex fluorescent imaging system (ProteinSimple, CA).

**Figure 1.**
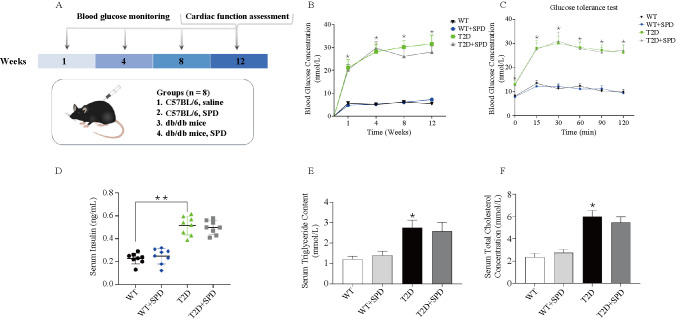
**Successful establishment of type 2 diabetic mouse model.** db/db mouse was selected as the research object of the type 2 diabetes model, and the related indexes were determined on 12 weeks: (A) Scheme of the in vivo experiment; (B) blood glucose concentration of different time point; (C) insulin resistance test; (D) serum insulin concentration; (E) triacylglycerol; (F) total cholesterol. *P < 0.05 vs WT group (*n* ═ 8). WT: Wild type; SPD: Spermidine; T2D: Type 2 diabetes.

### Quantitative real-time polymerase chain reaction (qRT-PCR) analysis

 To determine mRNA expression of Pannexin-1, GRP94, GRP78, ATF-4, and Chop, the total RNA was extracted from myocardial tissue and cardiac fibroblasts using the Qiagen RNeasy mini kit. One microgram of total RNA was used to synthesize cDNA, a portion of which (2 µl, equal to 0.4 µg cDNA) was used in a PCR with two appropriate primers (Table S1). The mRNA levels of target genes were detected using the miScript SYBR Green PCR kit (Qiagen) on CFX96 Touch Real-Time PCR system (Bio-Rad, MA, USA). ΔΔCT method was used to quantify all the relative mRNA levels using GAPDH as a reference and an internal standard for quantitation.

### Ethical statement

The study was approved by the Mudanjiang Medical University (MMU) Medical Science Ethics Committee (20220516-17).

### Statistical analysis

All experiments were replicated at least three times independently. All data were expressed as mean value ± standard error of mean (SEM). Statistical analysis was performed by two-tailed Student t-test or one-way ANOVA, followed by the Bonferroni multiple comparison test using GraphPad Prism 9.0. *P*-value < 0.05 was considered statistically significant.

## Results

### Establishment of type 2 diabetic mouse model

To better observe the effects of SPD, we selected 8-week-old wild-type mice and db/db mice as our research objects, and intraperitoneally injected with SPD every other day for 12 weeks, respectively. Glucose levels, glucose intolerance, insulin levels, triglyceride (TG), and TC levels were examined on week 12, which recapitulated the hallmark features of type 2 diabetes. The results showed that compared with the WT group, the blood glucose levels and glucose intolerance at each time point were higher, the insulin levels were significantly increased, and TG and TC were also increased in the T2D group and T2D+SPD group, Interestingly, compared with T2D group, there were no differences of the above indexes in the T2D+SPD group, also SPD had no effects in the WT group (Figure 1A–1F). It fully demonstrates that SPD will not affect the changes of the above indicators in mice.

### Exogenous SPD ameliorates cardiac functions in db/db mice

**Figure 2.**
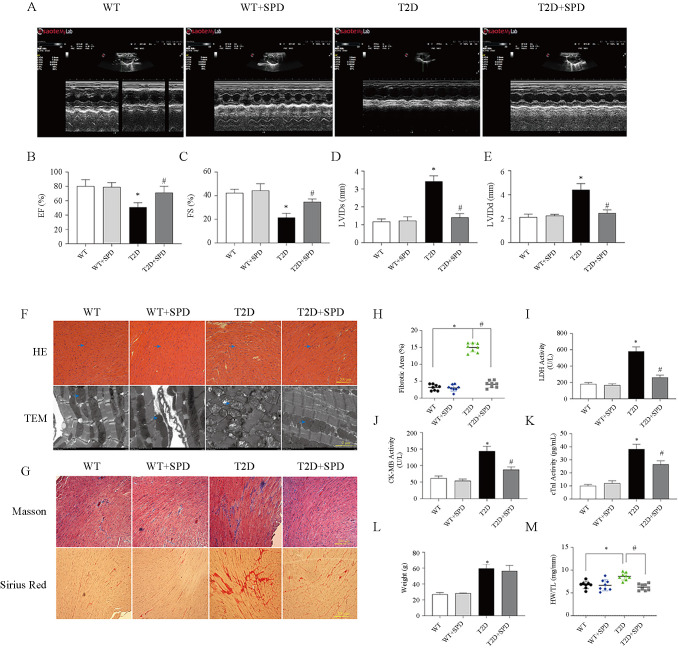
**SPD alleviates diabetic myocardial damage in db/db mice.** At week 12, the relevant indices of cardiac function were identified in C57BL/6 and db/db mice: (A) Echocardiography; (B) Left ventricular ejection fraction; (C) Left ventricular fractional shortening; (D) Left ventricular internal dimension systole; (E) Left ventricular internal dimension diastole; (F) Representative images of H&E staining and transmission electron microscopy; (G) Representative Masson’s trichrome and Sirius red staining of heart tissues; (H) Myocardial tissue fibrosis area statistics; (I) Serum lactate dehydrogenase concentration; (J) Serum CK-MB concentration; (K) Serum cTnI concentration; (L) Body weight; (M) The ratio of heart weight to tibia length. *P < 0.05 vs WT group; ^#^*P* < 0.05 vs T2D group (*n* ═ 8). SPD: Spermidine; CK-MB: Creatine kinase isoenzyme; cTnI: Cardiac troponin-I; WT: Wild-type; T2D: Type 2 diabetes.

To determine whether SPD can improve heart function in db/db mice, echocardiography was performed. Compared with the T2D group, we observed that EF and FS were increased, LVIDs and LVIDd were decreased in the T2D+SPD group, and WT mice treated with SPD had no obvious changes of cardiac functions (Figure 2A–2E).

To verify that DCM can cause serious cardiac injury, the hearts of mice in each group were excised in week 12, HE staining indicated that the cardiac myocytes were disordered and hypertrophic, TEM results revealed myocardial myofilament lysis and mitochondrial edema, and SPD could mitigate these pathological changes in db/db mice (Figure 2F). Masson and Sirius Red staining results showed large amounts of collagen deposition in the T2D group, and SPD could reduce collagen deposition (Figure 2G and 2H).

We also detected the serum myocardial injury markers (LDH, CK-MB, and cTnI) in each group. The results showed that the contents of serum LDH, CK-MB, and cTnI in the T2D group were significantly higher than those in the WT group. Compared with the T2D group, the serum contents of above enzymes in T2D+SPD group decreased significantly. These indicated SPD has protective and therapeutic effects on myocardial injury caused by high glucose (Figure 2I–2K). Moreover, body weight (BW) of db/db mice on week 12 was heavier than the WT mice BW, and SPD had no effects on BW. However, we found that SPD can reduce the ratio of heart weight to tibia length (HW/TL) in diabetic mice (Figure 2L and 2M).

**Figure 3.**
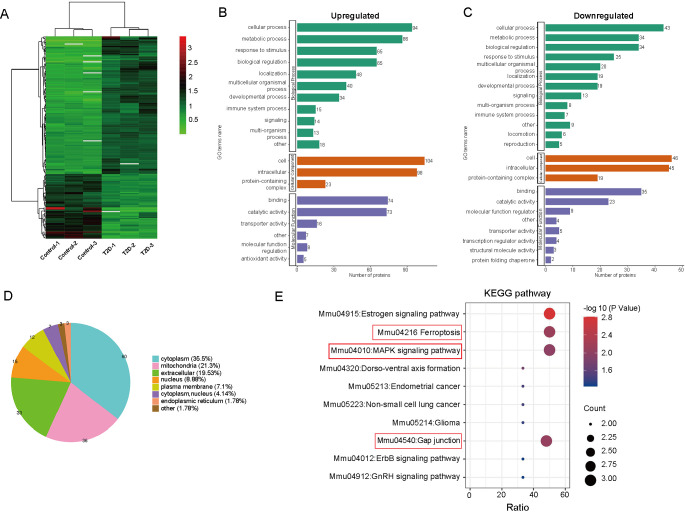
**Proteomic analysis of cardiac tissues from C57BL/6 mice and db/db mice:** (A) Heat map showing the quantitative protein expression in cardiac tissues from C57BL/6 mice and db/db mice; (B) and (C) Gene ontology analysis between C57BL/6 mice and db/db mice; (D) Subcellular distribution of the differentially expressed proteins of C57BL/6 and db/db mouse groups; (E) KEGG enrichment analysis for all the aberrantly expressed proteins, bubble diagrams displaying the top 10 KEGG pathways. The *Y*-axis and *X*-axis represent the names of the enriched pathways and the ratio of enrichment, respectively. The size of the bubbles indicates the number of differentially expressed proteins in each pathway. KEGG: Kyoto Encyclopedia of Genes and Genomes.

### Comparative proteomic analysis of cardiac tissues from WT mice and db/db mice

To further study the differential variations of protein expression in T2D mice, a liquid chromatography-tandem mass spectrometry (LC-MS) analysis of cardiac tissues in WT and db/db mice group was performed. Using the criteria fold change > 1.5 and *P*-value < 0.05, 169 proteins were identified as differentially expressed (DE) between the two groups (115 upregulated proteins and 54 downregulated proteins) (Figure 3A). The major GO terms including the related biological processes were identified, DE proteins were found to be enriched in cellular process, metabolic process, biological regulation, and response to stimulus (Figure 3B and 3C). Subcellular structure results showed that the largest proportion of DE proteins was identified in cytoplasma (35.5%), followed by mitochondria (21.3%), nucleus (8.88%), extracellular (19.53%), and plasma membrane (7.1%) (Figure 3D). DE proteins between WT and db/db mice group were mapped and enriched in KEGG pathways, the top 10 enriched pathways were shown in the functional KEGG enrichment cluster image (Figure 3E), we found that ferroptosis, mitogen-activated protein kinase (MAPK) signaling pathway, and gap junction were highly enriched in db/db mice compared to WT mice.

### Exogenous SPD restrains ferroptosis pathway in vivo and in vitro

**Figure 4.**
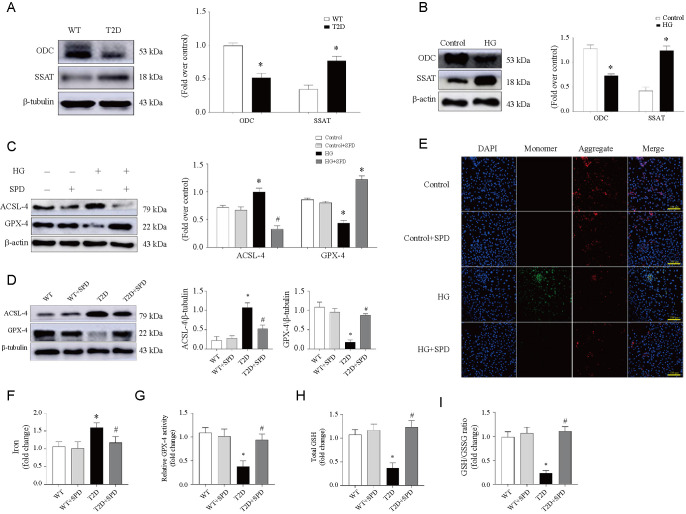
**SPD inhibits ferroptosis pathway in vivo and in vitro.** According to the analysis of proteomic results, diabetic cardiomyopathy is involved in ferroptosis pathway and related detections were performed: (A and B) Representative western blot of ODC and SSAT in comparison with β-actin expression in mice and cardiomyocytes; (C and D) The expression of ACSL-4 and GPX-4 in cardiomyocytes and mice; (E) Mitochondrial membrane potential (Δ ψm) was measured using JC-1 staining and photos were taken using fluorescence microscopy in cardiomyocytes treated with or without HG/SPD; (F–I) Heart tissues were collected in 12 weeks to measure iron, GPX-4 activity, GSH level, and GSH/GSSG ratio in the four groups. *P < 0.05 vs WT/Control group; ^#^*P* < 0.05 vs T2D/HG group (*n* ≥ 3). SPD: Spermidine; ODC: Ornithine decarboxylase; SSAT: SPD/spermine N1-acetyltransferase; GPX-4: Glutathione peroxidase 4; WT: Wild-type; T2D: Type 2 diabetes; GSH: Glutathione; HG: High glucose.

We detected the levels of pivotal PA metabolic enzymes in vivo and in vitro, the results showed that the cardiac expression of ODC was downregulated but SSAT was upregulated in db/db mice, and the expression trend is consistent with cell model results (Figure 4A and 4B). Now proteomics results have been verified that it is associated with ferroptosis, we then detected the key proteins of ferroptosis, and HG could upregulate ASCL-4 expression and downregulate GPX-4 expression in cardiomyocytes, treatment with SPD could gain the opposite results, which was consistent with the results of animal experiments (Figure 4C and 4D). Mitochondria is the key organelle of lipid metabolism, and abnormal lipid metabolism is closely related to mitochondrial damage [27]. JC-1 staining is often used as a tool to detect mitochondrial membrane potential (Δ Ψm). The result revealed that compared with the Control group, the red/green fluorescence intensity significantly decreased in the HG group, and the supplement of SPD increased the fluorescence intensity apparently (Figure 4E). In addition, we assayed iron, GPX-4 activity, GSH level, and GSH/GSSG ratio at 12 weeks in myocardial tissue. Iron, another essential factor for ferroptosis execution, whose activity was increased in diabetic mice, and SPD could decrease the activity significantly (Figure 4F). Subsequently, we demonstrated that the remaining three indicators had the opposite trend to iron activity (Figure 4G–4I).

### Exogenous SPD inhibits oxidative stress and endoplasmic reticulum stress in cardiomyocytes

**Figure 5.**
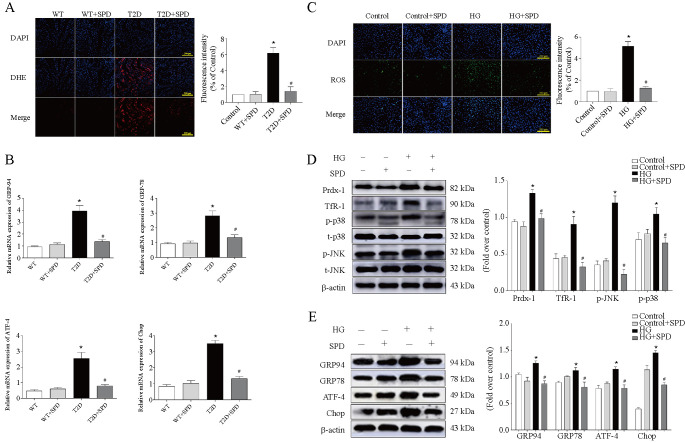
**SPD suppresses oxidative stress and endoplasmic reticulum stress in vivo and in vitro.** Mice and primary neonatal mouse cardiomyocytes were treated with or without SPD and underwent the following detections: (A) DHE staining of myocardial tissue; (B) mRNA expression of *GRP94, GRP78, ATF-4* and Chop; (C) Intracellular ROS level detection was determined by fluorescence microscopy; (D) Representative western blot of Prdx-1, TfR-1, p-p38, and p-JNK in cardiomyocytes; (E) The ER-related protein expression of GRP94, GRP78, ATF-4, and Chop was determined by western blot. *P < 0.05 vs Control group; ^#^*P* < 0.05 vs HG group (*n* ≥ 3). SPD: Spermidine; DHE: Dihydroethidium; ROS: Reactive oxygen species; ER: Endoplasmic reticulum; HG: High glucose

Excessive ROS deposition can activate ferroptosis-related pathways of cells by a series of cascade reactions [19, 28]. In our study, the DHE staining and ROS detection showed that SPD could reduce the oxidative stress levels in db/db mice and HG-treated cardiomyocytes (Figure 5A and 5C). Western blot results revealed that HG could upregulate Prdx-1, Tfr-1, p-p38, and p-JNK expression, and SPD could significantly downregulate the abovementioned proteins in cardiomyocytes (Figure 5D). Research demonstrated that ERS could induce ferroptosis via activation of ATF4-CHOP pathway [19]. We demonstrated that the mRNA expression of GRP94, GRP78, ATF4, and CHOP were upregulated in the heart tissue of the T2D group and SPD could downregulate above genes (Figure 5B). We then observed that the expression of GRP94, GRP78, ATF4, and CHOP increased in the HG group, and these protein expressions significantly decreased in the HG+SPD group (Figure 5E).

### Pannexin-1 regulates the P2X7 expression level in HG-treated cardiomyocytes

**Figure 6.**
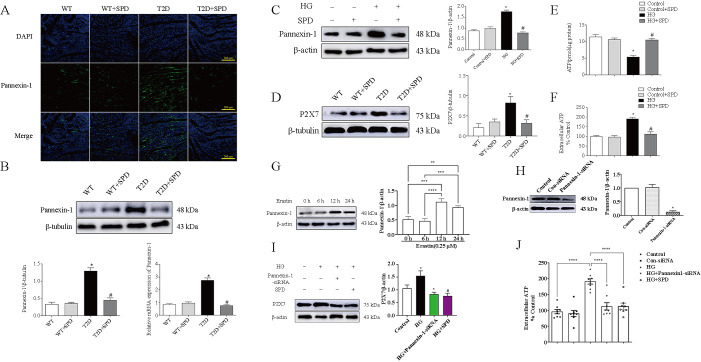
**The expression of P2X7 were regulated by Pannexin-1 in vivo and in vitro**: (A) The expression of Pannexin-1 in myocardial tissue was detected by immunofluorescence in C57BL/6 and db/db mouse; (B) The expression of Pannexin-1 in myocardial tissue were evaluated by western blot and qRT-PCR; (C) The expression of Pannexin-1 in cardiomyocytes treated with or without HG/SPD was determined by western blot; (D) The P2X7 expression in myocardial tissue was detected by western blot in C57BL/6 and db/db mouse; (E) The concentration of intracellular ATP was detected by chemiluminescence in cardiomyocytes treated with or without HG/SPD; (F) ATP concentration were determined in the medium of cardiomyocytes treated with or without HG/SPD; (G) Cardiomyocytes were treated with erastin and Pannexin-1 expression was detected in different time point; (H) Representative western blot of Pannexin-1 in comparison with β-actin expression; (I) Representative western blot of P2X7 in comparison with β-actin expression in cardiomyocytes; (J) ATP concentration was detected in the medium of cardiomyocytes treated with HG, SPD, and Pannexin-1–siRNA. *P < 0.05 vs WT/Control group/Control-siRNA; ^#^*P* < 0.05 vs T2D/HG group (*n* ≥ 3). qRT-PCR: Quantitative real-time polymerase chain reaction; SPD: Spermidine; ATP: Adenosine triphosphate; WT: Wild-type; T2D: Type 2 diabetes; HG: High glucose.

Immunofluorescence results showed that compared with the WT group, the Pannexin-1 fluorescence intensity significantly increased in the T2D group, and significantly decreased in the T2D+SPD group compared to the T2D group (Figure 6A), which was consistent with the trend of western blot and qRT-PCR results (Figure 6B and 6C). Hereafter, purinergic receptor P2X7 was determined in the heart tissue, and P2X7 expression was upregulated in the db/db mice, indicating more ATP emerged in myocardium. Notably, SPD could significantly inhibit P2X7 expression (Figure 6D). We also observed that HG could increase the levels of extracellular ATP and decrease intracellular ATP levels in cardiomyocytes, treatment with SPD could reverse the abovementioned changes (Figure 6E and 6F). Recently, it has been reported that ferroptosis is closely related to Pannexin-1 [29], and we used erastin (a special ferroptosis inducer) to treat with cardiomyocytes at different time points, and the results showed that erastin could upregulate the Pannexin-1 expression, which indicated that Pannexin-1 was involved in ferroptosis in cardiomyocytes (Figure 6G). Further experiments confirmed Pannexin-1–siRNA and SPD could both downregulate P2X7 expression level and extracellular ATP level in HG-treated in cardiomyocytes (Figure 6H–6J).

**Figure 7.**
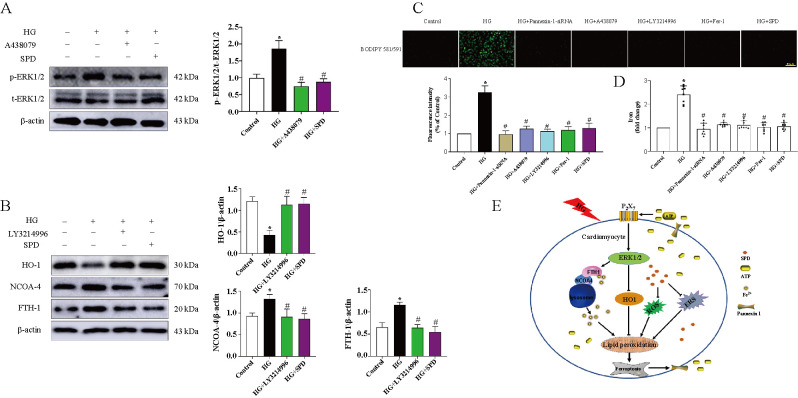
**SPD restrains ferroptosis via inhibiting ERK1/2–HO-1 pathway.** Cardiomyocytes were treated with HG, SPD, Pannexin-1–siRNA, A-438079, LY3214996, Fer-1 and underwent the following detections: (A) The ERK1/2 signaling pathway related protein expression was determined by western blot; (B) The expression of HO-1, NCOA-4, and FTH-1 were evaluated by western blot; (C) Intracellular lipid ROS was stained by BODIPY probe; (D) Iron content level of cardiomyocytes; (E) Schematic diagram showing the mechanism of SPD alleviating diabetic cardiomyopathy. *P < 0.05 vs Control group; ^#^*P* < 0.05 vs HG group (*n* ≥ 3). SPD: Spermidine; ROS: Reactive oxygen species; HG: High glucose; HO-1: Heme oxygenase 1; NCOA-4: Nuclear receptor coactivator 4; FTH-1: Ferritin light chain 1.

### Exogenous SPD suppresses ferroptosis via inhibiting ERK1/2–HO-1 pathway

To further study the mechanism of SPD alleviating DCM, we used P2X7 specific inhibitor (A-438079) and ERK1/2 signaling pathway blocker (LY3214996) to treat with cardiomyocytes separately. The results showed that HG could increase p-ERK1/2 expression and had no effect on t-ERK1/2 expression, indicating that it can activate phosphorylated ERK1/2 to activate the pathway. Conversely, A-438079 and SPD could significantly reduce its expression (Figure 7A). Furthermore, we demonstrated that HO-1 expression was downregulated, the expression of NCOA-4 and PTH-1 were upregulated in the HG group, and treatment with LY3214996 or SPD could obtain the opposite trend (Figure 7B). BODIPY probe results showed that HG could significantly increase the intracellular lipid ROS level and treatment with Pannexin-1–siRNA, A-438079, LY3214996, Fer-1, and SPD had markedly opponent effects (Figure 7C). In addition, iron assay showed that the variation tendencies of iron were consistent with the BODIPY probe results (Figure 7D).

## Discussion

In the present study, we selected db/db mouse, a model of type 2 diabetes as the research object, whose genetic background is C57BL/6 mouse diabetes gene knocked out and even insulin intervention fails to control hyperglycemia in db/db mouse. Therefore, db/db homozygous mice are widely used in the study of endocrine defects, neurological diseases, and cardiac diseases induced by abnormal glucose and lipid metabolism [30, 31]. At 1, 4, 8, and 12 weeks, db/db mouse showed persistent hyperglycemia, and there was no downward trend after SPD injection. At 12 weeks, insulin activity increased significantly in the T2D group, and serum TC and TG content levels increased significantly, the result was consistent with trends in insulin resistance test, which indicated that the type 2 diabetes mouse model was successfully established, however, SPD had no effects on the above indicators.

At 12 weeks, echocardiography displayed that EF and FS were decreased, LVIDs and LVIDd were increased in the T2D group, indicating heart systolic dysfunction. Further myocardial injury marker enzyme test confirmed that LDH, CK-MB, and cTnI levels were increased in the blood serum, and SPD could attenuate the above indicators of heart damage. In the T2D group and T2D+SPD group, db/db mice showed a significant increase in body weight, however, treatment with SPD could decrease the HW/TL ratio and we suspected it was due to remodeling of the heart and increasing the myocardial extracellular matrix. This speculation is supported by cardiac morphology. In the T2D group, Masson and Sirius red staining showed large amounts of collagen deposition in the interstitial areas, TEM results presented obvious myofilament dissolution and mitochondrial edema, HE staining displayed db/db mouse heart eventuated degeneration and necrosis of cardiomyocytes, and SPD could inhibit these changes effectively. These results demonstrated that SPD could alleviate high glucose-induced myocardial damages.

To further explore the mechanism of DCM in db/db mice, we used proteomics to analyze the differential expression of proteins in cardiac tissue between C57BL/6 and db/db mouse, and enrich in KEGG pathways. The results showed that DCM was closely related to ferroptosis pathway, MAPK signaling pathway, and gap junction, which indicated the next research direction of our project.

ACSL-4 and GPX-4 are the central regulators of ferroptosis, and they are often used as a marker of ferroptosis [32, 33]. In vivo, western blot results showed that ODC and ACSL-4 expressions were downregulated and SSAT and GPX-4 expressions were upregulated significantly in the T2D group, the opposite trend appeared in the T2D+SPD group, which was consistent with the proteomics results, and then we also observed the same variation tendency in vitro. Excess Fe2+ and cysteine deprivation are often linked to mitochondrial membrane hyperpolarization and lipid peroxide accumulation, leading to ferroptosis [34], In our study, mitochondrial membrane potential (Δ Ψm) increased markedly in the HG group, and treatment with SPD could reduce the above index. Moreover, increase of Iron level and decrease of GPX-4 activity, GSH level, and GSH/GSSG ratio in db/db mice could also demonstrate that ferroptosis was involved in DCM, and SPD could significantly reverse the abovementioned changes.

Oxidative stress causes disruptions in signaling pathways and oxidizes cellular components. As a result, the affected cells may become dysfunctional or undergo cell death through necrosis or apoptosis. Mitochondrial overproduction of ROS is the primary cause of damage, triggering increased polyol pathway flux, increased formation of advanced glycation end products (AGEs), and activation of the receptor for AGEs (RAGE) in DCM [35]. Oxidative stress damage is proposed as a main inducer of ferroptosis [36], and Prdx1 is a member of the Prdxs (peroxiredoxins) family and has been implicated as an antioxidant and redox signaling protein, acting as an antioxidant effect, and its expression is promoted when cells undergo oxidative stress [28, 37]. TfR1 is an important iron regulatory protein, mediating most of the cellular iron uptake by binding iron transferrin at the cell surface, which is internalized by receptor-mediated endocytosis, permitting the release and reduction of the iron in endosomes and transport of the released iron into the cytosol, and ferroptosis agonist can upregulate its expression to active p38 and JNK signaling pathway [38]. Our findings demonstrated that HG could increase ROS level, the expression of Prdx1, TfR1, p-p38, and p-JNK in cardiomyocytes, and SPD had the opposite effects. Recent studies have shown that ERS is involved in ferroptosis and aggravates cardiomyocytes injury [19], subsequently, we observed that GRP94, GRP78, ATF-4, and Chop expression were upregulated in the HG group and downregulated in the HG+SPD group.

Pannexin-1 belongs to the ATP-releasing pathway family and its function is the regulation of ATP flow out of the cell [39]. By immunofluorescence, we observed Pannexin-1 expression significantly increased in the T2D group and decreased in the T2D+SPD group, fitting well with the expression trends that we have observed with the in vitro experiments. P2X7 receptor is a family of purinergic G protein-coupled receptors, localizes to the cell membrane, and is stimulated by ATP [40], we found that P2X7 upregulated in the heart tissue of db/db mouse, which intimated the increasing leakage of endogenous ATP and treatment of SPD could reduce the expression of P2X7. To verify whether there was ATP leakage, we detected the endogenous and exogenous ATP concentration of cardiomyocytes, and the changes in the content of the two showed an opposite trend. That is to say, the endogenous ATP was markedly decreased in the HG group, but exogenous ATP was significantly increased, and the opposite tendency was observed in the HG+SPD group. Subsequently, we used erastin (ferroptosis agonist) to treat with cardiomyocytes, the expression of Pannexin-1 emerged time-dependent, indicating ferroptosis could promote its expression. Furthermore, we also observed that both Pannexin-1–siRNA and SPD could downregulate P2X7 expression and reduce the concentration of the exogenous ATP in the cardiomyocyte culture medium. Altogether, the abovementioned results further elucidated that HG could induce ferroptosis and activate Pannexin-1 expression, resulting in more leakage of ATP in cardiomyocytes and SPD appeared effective protection.

Our proteomic analyses have confirmed that MAPK signaling pathway is involved in the pathological process of DCM, and previous studies have shown that ferroptosis could activate the MAPK pathway through ERK1/2 [41, 42], we demonstrated that A-438079 (a specific inhibitor of P2X7) could restrain ERK1/2 signaling pathway, and SPD had the same effect. Research has found that heme oxygenase-1 (HO-1), nuclear receptor coactivator 4 (NCOA-4), and ferritin light chain 1 (FTH-1) were involved in regulating ferroptosis [43–47]. Western blot analysis showed that HG could suppress the HO-1 expression and promote NCOA-4 and FTH-1 expression. Conversely, treatment with SPD and LY3214996 (ERK1/2 signaling pathway inhibitor) had the opposite effects. Finally, both BODIPY probe and iron assay results certified that treatment with Pannexin-1–siRNA, A-438079, LY3214996, Fer-1, and SPD had the inhibition effects of HG-induced ferroptosis. Although our study confirmed the hypothesized mechanism in vivo and in vitro, further clinical patient data are needed for validation, which is also the obstacles of clinical translation.

## Conclusion

Taken as a whole, based on the aforementioned experimental results and present literature, it is hypothesized that in db/db mouse, persistent hyperglycemic stimulation leads to cardiomyocyte damages (ROS and ERS) and activation of ferroptosis pathway, and then upregulates the Pannexin-1 to open ATP outflow channel, resulting in an increase of extracellular ATP. Subsequently, increased ATP as a paracrine molecule combines to P2X7 receptors to activate ERK1/2 signaling pathway which regulate NCOA-4-mediated ferroptinophagy and antioxidant gene HO-1 expression. Conversely, SPD can reverse the above molecular regulation process (see Figure 7E). Hopefully, our results could provide novel targets and an experimental basis for the prevention and treatment of diabetic cardiomyopathy.

## Supplemental Data

**Table S1:** Nucleotide sequences of the primers used for real-time PCR

**Genes**	**Forward (5′–3′)**	**Reverse (5′–3′)**
*GAPDH*	GAAGGTGAAGGTCGGAGTC	GAAGATGGTGATGGGATTTC
Pannexin-1	GCTGTGGGCCATTATGTCTT	GCAGCCAGAGAATGGACTTC
*GRP94*	GGATGGTCTGGCAACATGGA	CCGAAGCGTTGCTGTTTCAA
*GRP78*	GACCCTTACTCGGGCCAAATT	GTAGAGCGGAACAGGTCCATGT
*ATF-4*	TCAGACACAGGCAAGGAGGA	GAACAGGGAAGAGGCTGCAAGA
Chop	ACGGAAACAGAGTGGTCAGT	AGACAGACAGGAGGTGATGC
